# High-density Genotyping reveals Genomic Characterization, Population Structure and Genetic Diversity of Indian Mithun (*Bos frontalis*)

**DOI:** 10.1038/s41598-018-28718-x

**Published:** 2018-07-09

**Authors:** Anupama Mukherjee, Sabyasachi Mukherjee, Rajan Dhakal, Moonmoon Mech, Imsusosang Longkumer, Nazrul Haque, Kezhavituo Vupru, Kobu Khate, I. Yanger Jamir, Pursenla Pongen, Chandan Rajkhowa, Abhijit Mitra, Bernt Guldbrandtsen, Goutam Sahana

**Affiliations:** 1 Animal Genetics and Breeding Lab., ICAR-National Research Centre on Mithun, Medziphema, Nagaland 797106 India; 2 Center for Quantitative Genetics and Genomics, Department of Molecular Biology and Genetics, Aarhus University, 8830 Tjele, Denmark; 3 Present Address: Dairy Cattle Breeding Division, ICAR-National Dairy Research Institute, Karnal, Haryana 132001 India

## Abstract

The current study aimed at genomic characterization and improved understanding of genetic diversity of two Indian mithun populations (both farm, 48 animals and field, 24 animals) using genome wide genotype data generated with Illumina BovineHD BeadChip. Eight additional populations of taurine cattle (Holstein and NDama), indicine cattle (Gir) and other evolutionarily closely related species (Bali cattle, Yak, Bison, Gaur and wild buffalo) were also included in this analysis (N = 137) for comparative purposes. Our results show that the genetic background of mithun populations was uniform with few possible signs of indicine admixture. In general, observed and expected heterozygosities were quite similar in these two populations. We also observed increased frequencies of small-sized runs of homozygosity (ROH) in the farm population compared to field mithuns. On the other hand, longer ROH were more frequent in field mithuns, which suggests recent founder effects and subsequent genetic drift due to close breeding in farmer herds. This represents the first study providing genetic evidence about the population structure and genomic diversity of Indian mithun. The information generated will be utilized for devising suitable breeding and conservation programme for mithun, an endangered bovine species in India.

## Introduction

Mithun (*Bos frontalis*), also known as gayal, a unique bovine species, has a limited geographical distribution primarily restricted to the North-Eastern Hilly (NEH) region of India, Myanmar, hilly provinces of Bangladesh, Bhutan and Yunan province of China^1^. Though it is difficult to ascertain the actual population of Mithun in the world, India possesses 0.30 million mithun which constitutes ~97.57% of the world population (19^th^ Livestock Census, 2012)^2^. Mithun has a very special socio-cultural status among the indigenous tribal population of NEH region of India. Having the very high value in the barter system, owning mithun is considered as a sign of prosperity.

Mithun are traditionally raised as a meat animal in a free-range system in the sub-tropical rain forest with almost zero input. As a part of socio-cultural practices, mithuns are sacrificed during rituals and festivals to offer feast^3^. Mithun meat is one of the most preferred sources of animal protein among local tribes. Compared to the meat from local cattle and buffalo, mithun meat is preferred due to both quality (better marbling, finer texture, and tenderness) and quantity (higher dressing %)^4–6^. Mithun have higher feed conversion efficiency than local cattle^7^. The current population size of mithun in India is not large. However, considering its importance in widening the biodiversity base and adaptation to a humid sub-tropical climate and a hilly topography, it could be exploited as an alternative means of livelihood as well as for improved food and nutrition security in its present habitat and in similar environments elsewhere.

Genetic improvement of mithun, however, is impeded by the way mithun is presently reared. Under traditional system of rearing, mithuns are let loose in the forest with minimal intervention. Recording of traits and pedigree information is almost non-existent. The size of a typical mithun herds ranges from 50 to 100. Herds are generally served by a few dominant bulls. The mithun population may therefore suffer from inbreeding. Under these circumstances, the genetic diversity of the mithun population needs to be described. This will assist ongoing mithun conservation and genetic improvement programme and for devising a suitable breeding policy.

Among a wide range of molecular markers developed, single nucleotide polymorphisms (SNPs) are the most abundant, widely dispersed throughout genomes, and have variable distribution among species^8^. The availability of high-throughput SNP genotyping platforms makes it feasible to undertake high-resolution scans by using large numbers of SNP markers distributed across the whole genome. SNPs are useful in studying livestock genetic diversity and population structure^8,9^. Although a large number of SNPs have been identified in bovine genome-sequencing projects, few of these have been validated outside *Bos taurus*, as for example in mithun^10^. The Illumina BovineHD BeadChip (Illumina, San Diego, CA) with 777k SNPs was introduced and utilized for genotyping studies in various breeds of cattle, but has also been used in other members of the bovidae, for example, yak, gaur, buffalo and wild anoa^10–16^.

Other bovine species including indigenous cattle, yak and gaur are also found in the North Eastern parts of India along with mithun. Forest cover and natural habitats of mithuns in the North Eastern Hill States of India have shrunk over the years. This raises concerns about possible introgression of local cattle with mithun, sharing the same habitat. Sporadic instances of crossing mithuns with cattle bulls by mithun owners in the field to increase milk production have also been recorded^17^. Hence, we used the unsupervised clustering analysis (carried out with the ADMIXTURE software)^18^ to estimate individuals’ ancestries from SNP genotypes to assess the extent of admixture in mithun with other cattle species (Table 1).

**Table 1:** Species/breeds included in the ADMIXTURE and PCA analyses genotyped with Illumina BovineHD BeadChip (HD) or BovineSNP50 (50 k).

Sl. No.	Name/Code used	Location of collected samples	Sample size	
			Admixture analysis	PCA analysis
1	Mithun – farm population (*Bos frontalis*)/Farm	India	48	20
2	Mithun – field population (*Bos frontalis*)/Field	India	24	20
3	NDama (*Bos taurus*)/NDA	Africa	23	20
4	Bali cattle (*Bos javanicus*)/BLI	Indonesia (Bali)	20	20
5	Gir (*Bos indicus*)/ GIR	Brazil	50	20
6	Holstein (*Bos taurus)/*HOL	Denmark	20	20
7	Wild buffalo (*Bubalus depressicornis*)/OWB	Africa	10	10
8	Yak (*Bos grunniens*)/OYK	China	4	4
9	Bison (*Bison bison*)/OBB	USA	4	4
10	Gaur (*Bos gaurus*)/OGR	USA	6	6

Therefore, the present study aimed to generate high-resolution information on the genomic diversity and population structure of mithun using Illumina BovineHD BeadChip. To our knowledge, this is the first study using BovineHD BeadChip in mithun. We further studied the evolutionary relationship of mithun with closely related bovine species to understand mithun phylogeny and origin.

## Results

### Population diversity parameters - Observed H_o_, expected H_e_, F_IS_ and ROH

Observed (H_o_) and expected (H_e_) heterozygosities estimated in the Indian mithun population (farm and field population) ranged from 0.25 to 0.17, and 0.25 to 0.18, respectively (Table 2). The inbreeding coefficient estimates (F_IS_) based on the observed versus expected number of homozygous genotypes, were found to be 0.06 ± 0.02 and 0.02 ± 0.01 for the farm and field animals (Table 2). The mean F_ST_ estimate between farm and field mithun populations was 0.03 ± 0.01.

**Table 2:** Geographic location, sample size (N), composition of mithuns and diversity parameters in mithun population.

Population	Location	No. of animals	Average H_o_	Average H_e_	Inbreeding Coefficient (F_IS_)
Research- farm (Farm)	Nagaland	48 (28 males and 20 females)	0.25 ± 0.08	0.25 ± 0.07	0.06 ± 0.02
Farmers’ herds (Field)	Nagaland, Arunachal Pradesh, Manipur, Mizoram	24 (11 males and 13 females)	0.17 ± 0.03	0.18 ± 0.03	0.02 ± 0.01

Average observed and expected heterozygosities are indicated as H_o_ and H_e_, respectively.

### Runs of homozygosity

Runs of homozygosity (ROH) in the autosomes of 48 farm mithun and 24 field mithun animals were determined using PLINK1.9^19^ and consisted of 139,350 SNPs in each of the farm and field data set after quality control.

The total length of ROH per animal was averaged within population in six sized windows: 250 kb–1 Mb, 1 Mb–2 Mb, 2 Mb–4 Mb, 4 Mb–8 Mb, 8 Mb–16 Mb and >16 Mb. The average total length of ROH per animal was 823.7, 242.1, 92.30, 47.03, 66.22 and 110.9 Mb in the farm and 735.8, 245.0, 121.3, 84.4, 86.0 and 87.4 Mb in the field population for these six length categories.

**Figure 1:**
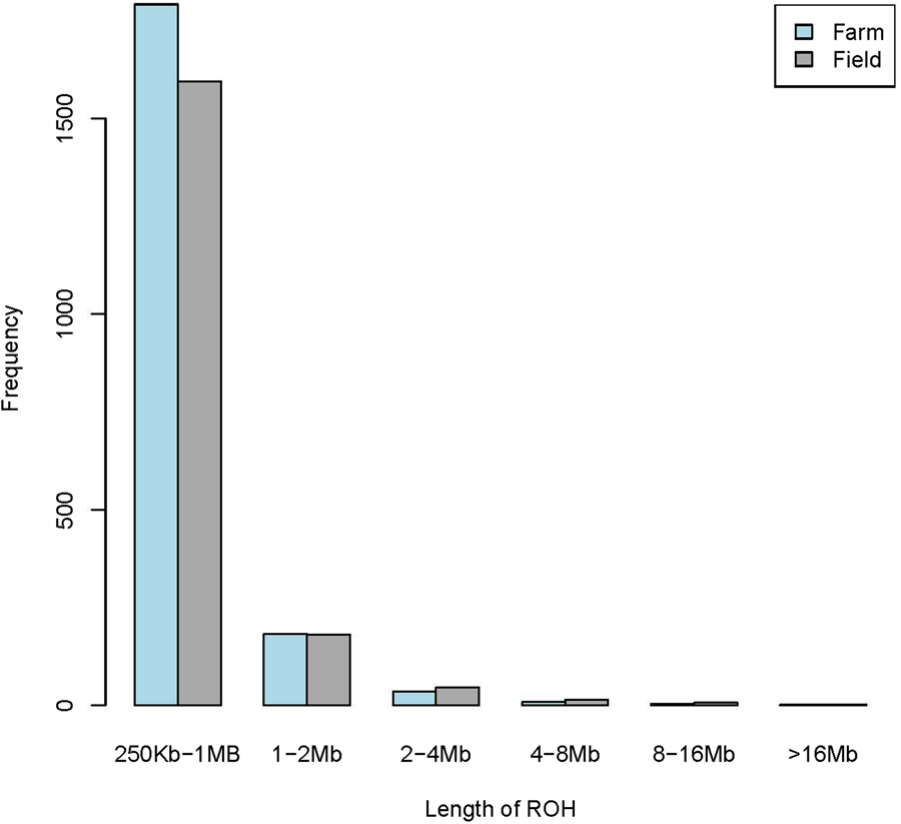
Average number of ROH for different lengths in farm and field mithuns.

Summary statistics of ROH observed are outlined in Table 3 (farm) and 4 (field). Average numbers of ROH per animal for these six categories were 1793.0, 182.8, 35.3, 8.9, 6.0 and 4.25 in farm and 1596.0, 181.9, 45.5 15.5, 7.7 and 3.6, respectively in field population. Patterns of ROH are roughly similar up to about 2 Mb of length corresponding to more than about 25 generations ago. For ROH between 2 and 16 Mb (roughly 25–26 generations ago) the field population has more ROH. Proportions of very long ROH are higher in the farm populations. This suggest an older history that may be shared between the farm and field populations, a period of relative depression of the effective population size for the field population, followed by a period of relatively higher close breeding in the farm population. The largest total length of autosomal ROH observed corresponded to approximately 27% of the genome length for farm and 23% for field animals. The average number of ROH of different lengths in farm and field mithuns are ploted to show their frequency distribution (Fig. 1).

**Table 3:** ROH and F_ROH_ in the farm mithun population.

	Statistics	ROH length category (Mb)					
		250 kb-1 Mb	1–2 Mb	2–4 Mb	4–8 Mb	8–16 Mb	>16 Mb
Length of ROH per animal (Mb)	Mean	823.7	242.1	92.30	47.03	66.22	110.9
	SD	85.3	42.1	39.3	34.2	54.6	134.7
	Min	610.7	137.8	25.31	4.52	8.2	16.7
	Max	971.6	322.8	179.8	138.1	205.6	498.6
Number of ROH per animal	Mean	1793.0	182.8	35.3	8.9	6.04	4.25
	SD	199.8	30.1	14.5	6.2	4.7	5.02
	Min	1313.0	108.0	10.0	1.0	1.0	1.00
	Max	2125.0	240.0	69.0	26.0	18.0	19.00
F_ROH_	Mean	0.335	0.098	0.038	0.019	0.027	0.045
	SD	0.035	0.017	0.016	0.014	0.022	0.007
	Min	0.248	0.056	0.010	0.002	0.003	0.007
	Max	0.395	0.131	0.073	0.056	0.084	0.202

**Table 4:** ROH and F_ROH_ in the field mithun population.

	Statistics	ROH length category (Mb)					
		250 kb-1 Mb	1–2 Mb	2–4 Mb	4–8 Mb	8–16 Mb	>16 Mb
Length of ROH per animal (Mb)	Mean	735.8	245.0	121.3	84.4	86.0	87.4
	SD	110.8	679.8	460.4	372.0	559.7	59.4
	Min	400.6	111.9	38.1	14.6	10.2	17.3
	Max	893.7	499.2	256.3	166.6	198.3	187.4
Number of ROH per animal	Mean	1596.0	181.9	45.5	15.5	7.7	3.6
	SD	235.7	48.9	17.1	6.7	5.0	2.4
	Min	894.0	82.0	15.0	3.0	1.0	1.0
	Max	1987.0	362.0	96.0	32.0	17.0	7.0
F_ROH_	Mean	0.294	0.099	0.049	0.034	0.034	0.035
	SD	0.045	0.027	0.019	0.015	0.022	0.024
	Min	0.007	0.045	0.015	0.005	0.004	0.007
	Max	0.076	0.202	0.104	0.067	0.080	0.076

### Admixture Analysis

Unsupervised clustering analysis using ADMIXTURE software program were carried out for 10 populations (total 209 samples; Table 1) including one indicine cattle breed, Gir and two taurine breeds (Holstein and NDama) to identify possible indicine and taurine introgression in the mithun population. The analysis was done using 50k markers.

**Figure 2:**
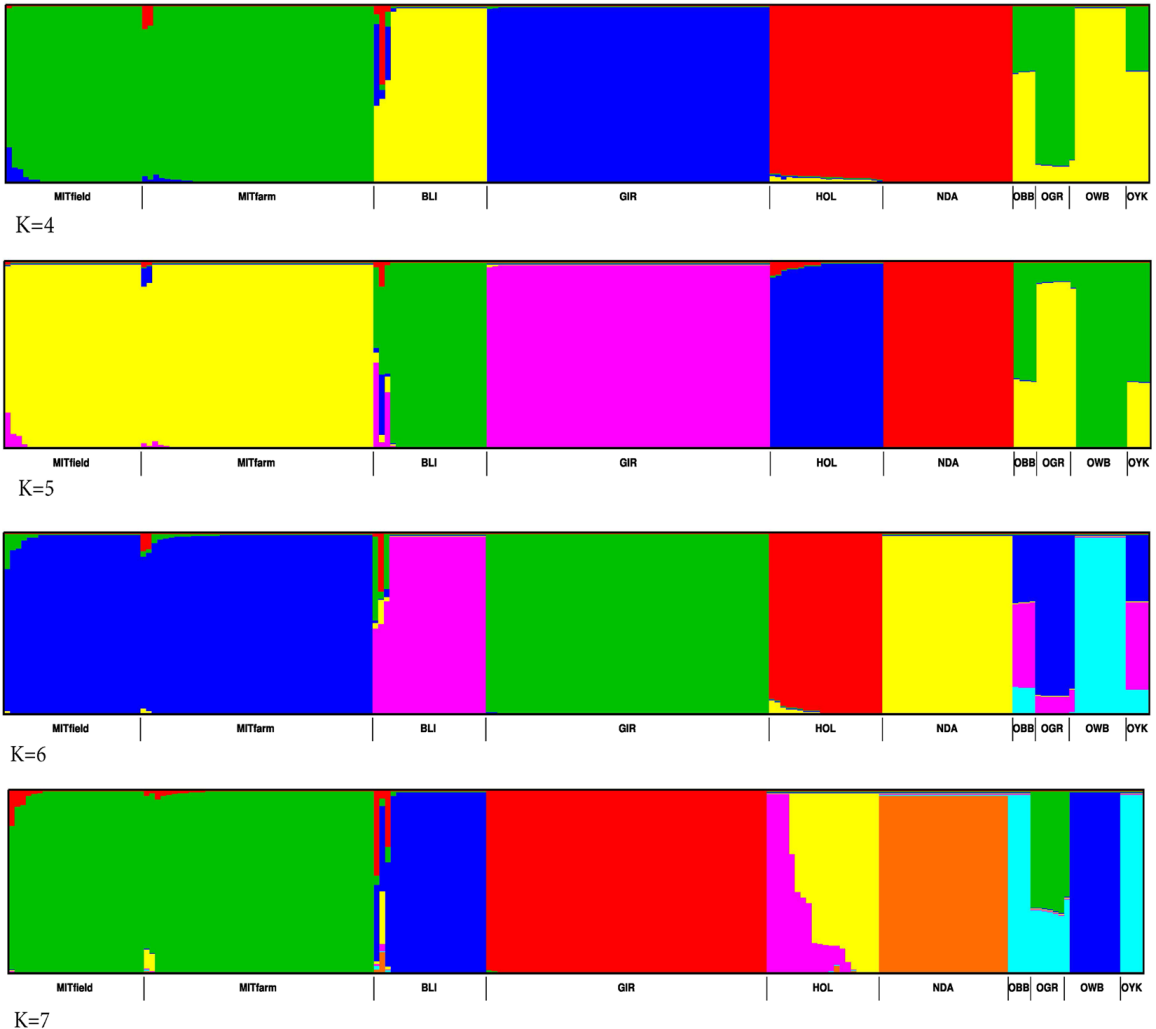
Plot of admixture analyses results for K = 4 to 7 using 38,587 autosomal SNP. K = 6 yielded the lowest cross-validation error. Abbreviations: MITfarm: Farm mithun, MITfield: Field mithun, BLI: Bali cattle, GIR: Gir cattle, HOL: Holstein, NDA: NDama; OBB: Bison; OGR: Gaur; OWB: Wild buffalo, and OYK: Yak.

**Figure 3:**
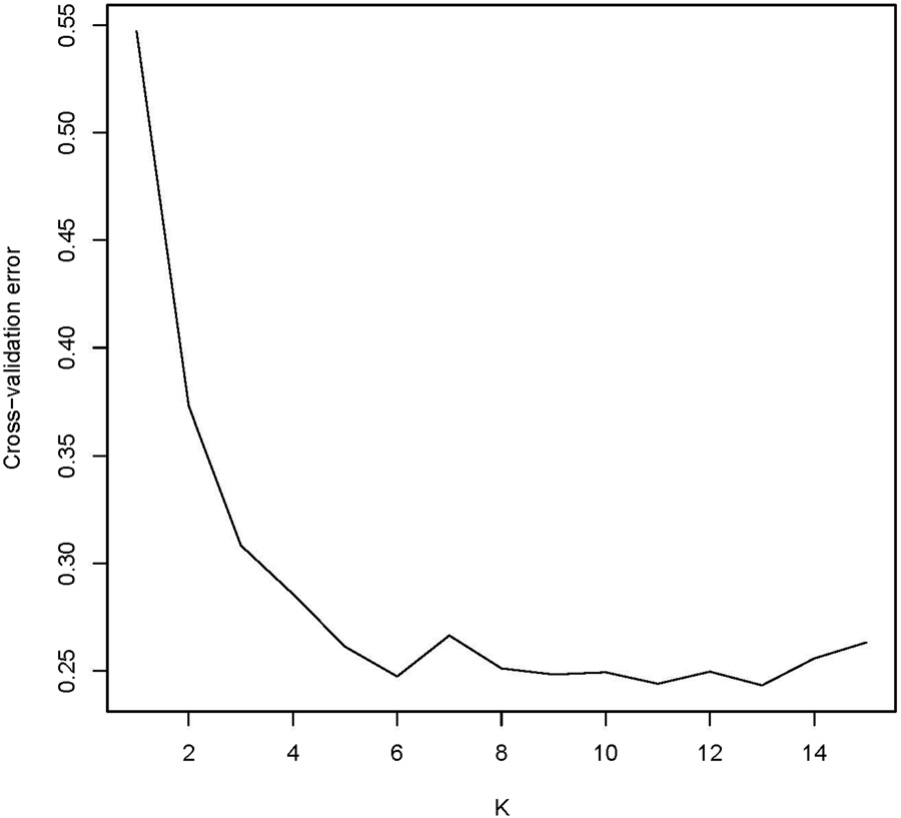
Cross-validation errors for the SNP dataset used for admixture analysis for varying numbers of population components (K).

The admixture analysis results of farm and field mithuns do not show any population structure (details not presented). The admixture analysis including other species grouped mithuns from farm and field together, and were fairly homogeneous (Fig. 2, K = 4–7). With K = 4, we observed a strong genetic differentiation between mithun (colored in green) and the remaining bovine species included in this study, except gaur (OGR), yak (OYK) and bison (OBB), which shared a common genetic component. At K = 7, mithun was distinguished from all other bovine species except gaur. There were minute levels of indicine and taurine introgression in the mithun population. A plot using cross-validation errors (CVE) was drawn for the SNP dataset used for admixture analysis (Fig. 3), showing overall uniform values of CVE beyond K = 6.

### Principal Component Analysis

**Figure 4:**
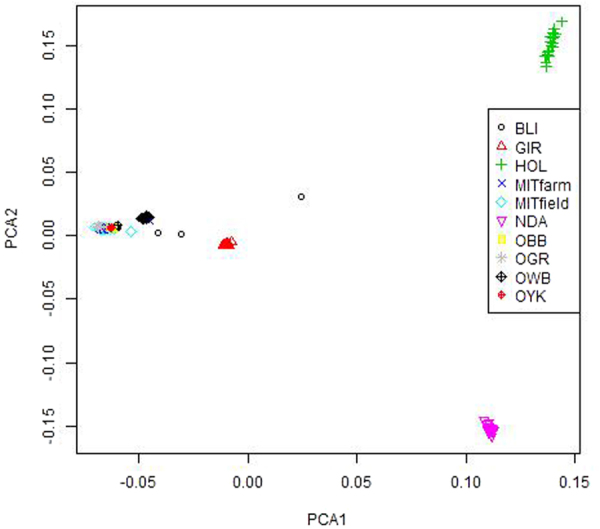
Principal components analysis of Indian mithun along with other bovine species based on autosomal SNPs. PCA1 and PCA2 explained 36.6% and 11.8% of the variance respectively. MITfarm: Farm mithun, MITfield: Field mithun, BLI: Bali cattle, GIR: Gir cattle, HOL: Holstein, NDA: NDama; OBB: Bison; OGR: Gaur; OWB: Wild buffalo, and OYK: Yak.

The principal component analysis was done using 50k markers for eight Bos species and two mithun populations (firm and field). The first two principal components (PC1 and PC2) grouped farm and field mithuns in a cluster with gaur, yak and bison, separated from three other major clusters of two taurine cattle (NDama and Holstein), and indicine cattle (Gir; Fig. 4).

**Figure 5:**
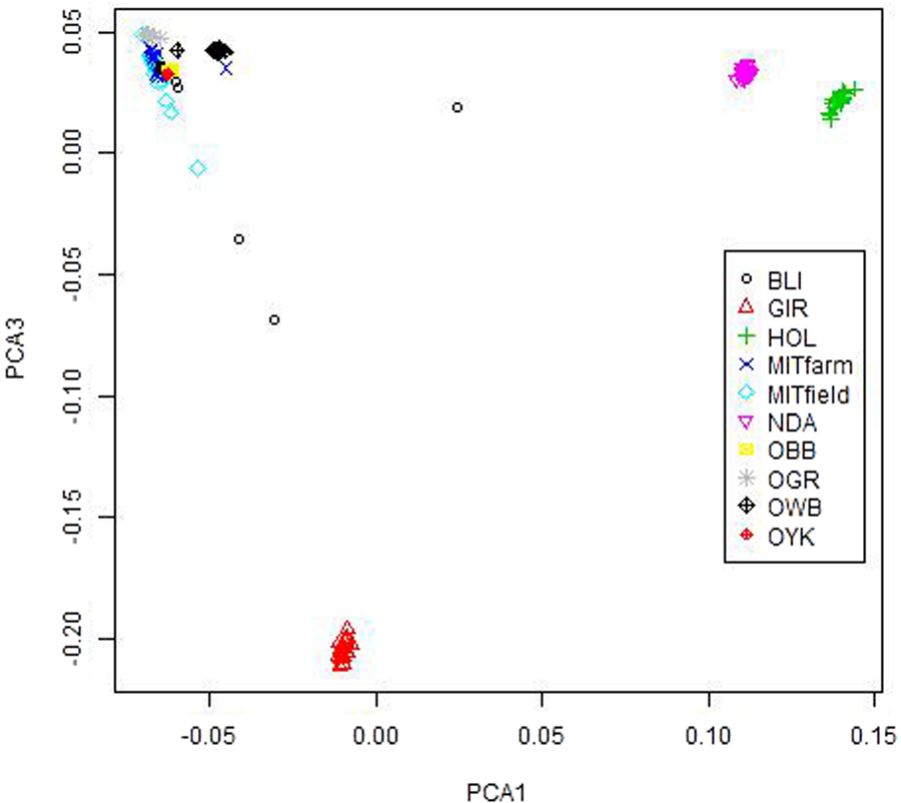
Plot of principal components 1 and 3 positioned Indian mithuns along with OGR: gaur, OYK: yak, and OBB: bison in distinct cluster from major taurine and indicine cattle populations. PCA1 and PCA3 explained 36.6% and 7.4% of the variance, respectively.

Principal component1 (PC1) positioned mithuns along with gaur, yak, bison and Bali cattle in distinct clusters from indicine and taurine cattle populations. Principal component 2 (PC2) separated two taurine breeds into separate clusters. Principal component 3 (PC3) further separated Gir from other populations (Fig. 5).

### Phylogenetic analysis using Treemix

**Figure 6:**
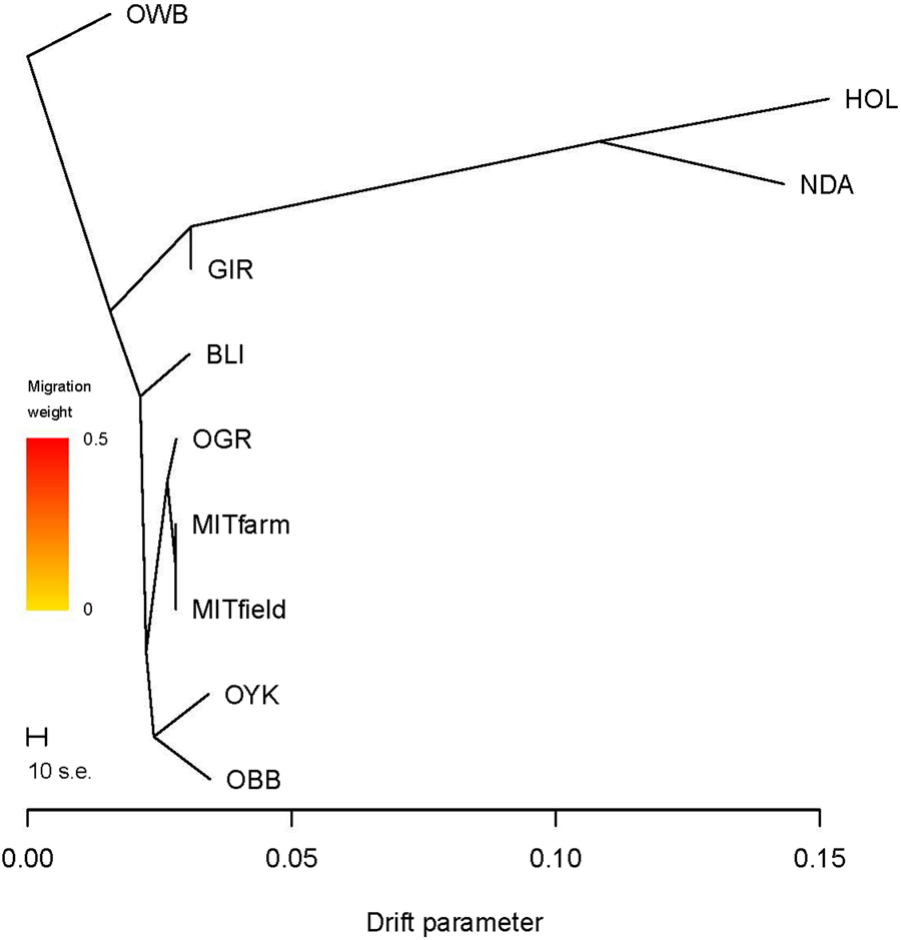
Phylogenetic tree to infer the position of mithun among eight bovine species. The bootstrap support for all the branching ranged between 99 and 100%.

Treemix^20^ analysis was used to study the population splits and subsequent gene flows. We constructed a phylogenetic tree of all the bovine species without adding any migrations and assuming wild buffalo (OWB) as outgroup using Treemix analysis (Fig. 6). Threepop reveals no evidence of admixture (the smallest test statistics was +2.76). We also analysed to fit 0–5 migrations in Treemix, but there was no evidence of migration (details not presented). While two mithun populations and gaur were in one single clade, the indicine breed, Gir, was in one group, two taurine breeds were in one group, Bali cattle was in a group by itself, and yak and bison formed another group. The taurine breeds Holstein and NDama animals formed a separate group. Gir, an indicine cattle breed, branched out from taurine as expected.

## Discussion

India has the largest mithun population (~97.57%) in the world (0.30 million, 19^th^ Livestock Census, 2012). Myanmar (0.96%, approx. 3000), Bangladesh (0.32%, approx. 1000), China (0.96%, approx. 3000) and Bhutan (0.18%, approx. 570) also home small populations of mithun in Asia^2^. Information regarding genetic diversity and population structure of Indian mithun remains scanty. A conservation programme for mithun has been initiated at Khonoma and Thevopisu villages, Nagaland in their native breeding tracts in India. Genetic improvement in mithun through a systematic breeding program could not be initiated under field conditions due to the small number of animals, uncontrolled mating, and their scattered distribution across remote and inhospitable hilly terrain.

Genetic diversity of two groups of Indian mithun population (farm and field groups) were assessed by expected heterozygosity (H_e_), observed heterozygosity (H_o_) and estimates of inbreeding (F_IS_ and F_ST_). F_ST_ between two populations was low (0.03) indicating a close genetic connection between them^21^. Mithun is assumed to be a close evolutionary relative of gaur^3,22,23^. Hence, the BovineHD BeadChip was used to genotype mithuns and available literature on cattle was referred for comparison in the present study. Genetic diversity in terms of average heterozygosity in both mithun populations (0.17–0.25) was found to be similar to zebu and African taurine cattle^14^, and was in the same range as for taurine cattle from West Africa (from 0.18 for African Lagune to 0.22 for Somba cattle) or East Africa (0.24 for Sheko cattle)^10^. However, SNPs that are included in the analyses are polymorphic within Mithun, but originate from bovine and are thus expected to be old SNPs (polymorphic in the common ancestor of Mithun and *Bos taurus*) leading to biased allele frequency spectrum.

We used F_IS_ as a measure to study inbreeding within farm and field mithun populations. Estimated F_IS_ values indicated low levels of inbreeding. F_IS_ was lower in the field (0.02) than in the farm population (0.06). We see some evidence of mating between relatives in the farm population, while there is little evidence of mating among close relative in the field population. The F_IS_ values in our study were similar to that reported^24^ in mithuns from Bangladesh using the Illumina BovineSNP50 BeadChip, mean expected heterozygosity of 0.148 ± 0.14 with a heterozygote deficiency of 0.06 (F_IS_).

Lower heterozygosity values H_o_ and H_e_ of mithun population in our study compared to taurine and indicine cattle breeds as reported by the Bovine HapMap Consortium^12,13^ and Korean cattle populations^16^ may also be attributed to ascertainment bias in SNP discovery of the BovineHD BeadChip. Previously low heterozygosity estimates of Tunisian cattle populations were likewise attributed to ascertainment bias in the design of the BovineSNP50 BeadChip as polymorphic sites of African origin present in the genome of Tunisian cattle were not included in the chip^14^. The Bovine HapMap Consortium reported that nucleotide diversity in (Brahman) cattle, a crossbred of indicine breeds, is more than twice that observed within Holstein and Angus breeds^25,26^ as expected.

Our study revealed a higher number of short and medium ROH (250 kb–1 Mb, 1–2 Mb, 2–4 Mb, and 4–8 Mb) than longer categories (8–16 Mb and >16 Mb) in both the farm and field mithun population. This reflects ancestors shared between the parents long ago, can be indicative of selective sweep, ancient inbreeding or bottleneck. The farm population showed greater ROH length and numbers compared to field population, which was as per expectation due to higher level of inbreeding and small population size parental population in the farm. Similar observations in various cattle breeds were reported as short ROH are generally due to older haplotype relatedness, while longer ROH result from more recent inbreeding^27–29^.

Length and frequency of ROH provide information about demographic history as well as recent inbreeding in individuals^27,30,31^. Long ROH indicates consanguinity between an individual’s parents^27^. Shorter ROH indicate more distant demographic and selective events after repeated fragmentation of chromosome segments by recombination^27^. In particular, recent inbreeding resulting from the mating of closely-related ancestors leads to a high occurrence of long ROH. On the other hand, very long ROH sometimes occur in outbred populations^32^.

In human, short ROH (<1.5 mb) is due to ancient linkage disequilibrium through inheriting parental common haplotypes, whereas, bigger ROH is due the history of related parents in recent times^33^. Level of inbreeding can be estimated accurately in cattle population without knowledge of the pedigree using Illumina BovineHD SNP genotyping assay^34^. F_ROH_, the genomic inbreeding coefficient, was found to be highly correlated with pedigree inbreeding in a small group of populations^30,34^.

Estimates of mean F_ROH_ for the various categories (250 kb–1Mb, 1 Mb–2 Mb, 2 Mb–4 Mb, 4 Mb–8 Mb, 8 Mb–16 Mb and >16 Mb) in the farm and field mithun population were generally low, probably indicating larger effective population sizes in the past. We observed relatively higher mean F_ROH_ for the 250kb–1Mb category in the farm mithun (0.335). Such short ROH reflect events at least 25 generations ago. With generation interval of approximately 5–10 years in mithun, this translates to selection 100 or 200 years in the past. It seems that probably mithun population has experienced a population bottleneck in the past.

Our results fit well with other genotyping studies in various breeds of cattle using Illumina BovineHD BeadChip and validated through Illumina BovineSNP50 BeadChip, viz. average autozygosity calculated from ROH (F_ROH_) with lengths above 1 Mb was in intensely selected Brown Swiss breed (0.151–0.156)^33,35^ and in Holstein cattle (0.116)^29^, while lower F_ROH_ for unselected cattle breeds Pinzgauer and Tyrolean Grey domestic cattle (0.062 and 0.066, respectively)^35^, and the lowest in unselected, preserved Polish Red breed (0.057)^29^.

We found Indian mithun studied here constitute a genetically uniform group. Influence from taurine and indicine cattle was comparatively minor. Admixture analysis detected a small proportion of admixture of Indian mithun with indicine or taurine cattle. However, based on Treemix analysis there was no evidence of direct gene flow to Indian mithun from cattle, indicating the local tribal practice of crossing mithun with cattle under the field conditions. This crossing probably was with a cattle population not closely related to the breeds represented in our study. Treemix results indicated there was considerable genetic similarity between Indian mithun and gaur, and consistently placing mithun and gaur in the same clade.

## Conclusion

To our knowledge, this is first study aiming to assess the genetic structure of Indian mithun and population diversity using the BovineHD BeadChip SNP array. Our results provide information about the genomic diversity, population structure and origin of Indian mithun inhabiting North Eastern Hilly region. We found evidence of admixture of taurine and indicine in some mithuns, signifying crossing with cattle under field conditions. We also showed that the Indian mithuns are having distinct genetic characteristics and common ancestry with gaur. There was a substantial amount of inbreeding detected as ROH, which has to be considered in future sustainable breeding and conservation programs for the species. Overall introgression from other bovine species into Indian mithun was limited. Our results are consistent with the mithun being domesticated from a population related to the extant gaur. Our results do not support the hypothesis that mithun originated from crossing gaur bulls with indigenous cattle.

Our study provides a comprehensive picture of the genetic structure and population diversity of Indian mithuns and their phylogenetic relationship with other bovine species.

## Methods

### Study Populations and sampling

The mithuns were selected randomly from a randomly mating population maintained at the ICAR-NRC on Mithun research farm, Medziphema, Nagaland (n = 48) and a diverse field population (n = 24) from four locations of North Eastern Hill Region (Nagaland, Arunachal Pradesh, Manipur and Mizoram) falling in the native mithun breeding tracts of India. All the experiments were performed in accordance with relevant guidelines and regulations approved by the Institutional Animal Ethics Committee, ICAR-NRC on Mithun, Nagaland.

### DNA processing and genotyping with BovineHD BeadChip (777 k)

#### DNA extraction

Blood samples were collected from jugular vein in a vacutainer tube (BD) containing EDTA by a qualified veterinarian and were transported to the laboratory in cool pack as soon as possible. The DNA was isolated using a standard blood DNA isolation kit (Promega #A1620) as per the manufacturer’s instructions.

#### Genotyping and Quality Control

Genomic DNA from each mithun was quantified to assure a concentration of at least 50 ng/µl genomic DNA required by the Illumina® Infinium® SNP genotyping platform. A total of 72 mithun (39 males and 33 females) were genotyped by Sandor Lifesciences Pvt. Ltd., Banjara Hills, Hyderabad, India with the Illumina® BovineHD BeadChip assay, following the manufacturer’s protocol. Genotypes were called using the validated standard cluster file provided by the manufacturer. Only the autosomal SNPs were considered in this study.

Samples and marker based quality control was performed using the Illumina’s Genome Studio software (https://support.illumina.com/array/arraysoftware/genomestudio/documentation.html). Genome Studio was also used to generate PLINK1.9 data files (in.*ped* and.*map* format)^19^ for further analyses. The SNPs located on sex chromosomes, not polymorphic or without known position in the cattle genome were excluded from further analysis. The *Bos taurus* genome assembly (UMD3.1; http://www.ensembl.org/Bos_taurus/Info/Index) was used as a reference genome due to the absence of a published mithun genome.

To identify closely related individual, a pair-wise identity-by-state (IBS) distance analysis was performed using PLINK1.9^19^. No closely related individuals were detected, based on a significance test criterion of whether two individuals belong to the same population (i.e. do not merge clusters that contain significantly different individuals). SNP markers showing deviation from Hardy-Weinberg proportions (HWP) based on parental genotype data (*p* < 0.001) were removed. The attributes considered for quality control finally include filtering of SNPs with call rate ≥95%, MAF ≥5% and HWP ≥0.001 using PLINK1.9 software. After this filtration 139,350 polymorphic SNPs remained for analysis.

#### Population genetics analysis

The parameters estimated to study the genomic diversity in mithun populations included estimates of heterozygosity, detection of runs of homozygosity (ROH) and estimates of inbreeding coefficients.

The observed (H_o_) and expected (H_e_) heterozygosities, as well as the estimates of inbreeding for mithun populations were estimated using PLINK1.9 software^19^. The F_ST_ was estimated using PLINK software, which uses method introduced by Weir and Cockerham (1984)^36^.

#### Detection of autozygosity in genomic region

Runs of homozygosity (ROH) were detected to determine the extent of autozygosity in the mithun populations. A ROH is defined as a contiguous length of homozygous genotypes. A ROH of sufficient length indicates the two copies of the chromosome in this region are identical-by-descent (IBD)^30^. The length and frequency of ROH is useful to get insights into the history of inbreeding of an individual and the population.

PLINK1.9^19^ was used to identify ROH by running a sliding window that scans the genomic distribution of SNP data to identify stretches of homozygous SNPs. A minimum number of 10 consecutive homozygous SNP and zero heterozygotes were allowed in each window. A maximum gap between SNP of 1,000 kb was allowed. With high-density SNP data this approach mostly detects truly autozygous segments^37^. Moreover, this strategy is particularly suitable for livestock populations because they have much higher levels of autozygosity than model organisms making identification of longer ROH easy^35,37^.

Detected ROH were classified in windows: ROH in the range of 250 kb-1 Mb, 1 Mb–2 Mb, 2 Mb–4 Mb, 4 Mb–8 Mb, 8 Mb–16 Mb and >16 Mb.

In the present study, genomic autozygosity, F_ROH_ of each individual was estimated as the sum of length of autosomal ROH divided by the total length of the autosomes covered by markers^34^.

#### Population structure and origin of Mithun

The main goal of the present study was genomic characterization of two mithun populations (farm and field). Another eight Illumina® BovineHD BeadChip or BovineSNP50 BeadChip genotype data sets were collected from previously published work, available at [http://widde.toulouse.inra.fr/widde/]^38^ and were included along with two mithun populations to explore the evolutionary relationship of mithun with other bovine species (Table 1). The wild gaur (*Bos gaurus*, OGR, n = 6) was included assuming contribution of gaur to mithun genomes as its presumed wild relative; while yak (*Bos grunniens*, OYK, n = 4), bison (*Bison bison*, OBB, n = 4) and Bali cattle (*Bos javanicus*, BLI, n = 20) were taken into account to find out possible genetic contribution towards mithun genome, if any. As it is assumed that there might be some introgression due to crossing between mithun and cattle under the field conditions, the indicine cattle, Gir (*Bos indicus*) genotype data was included. Taurine cattle (NDama, NDA, n = 23; Holstein, HOL, n = 20) were included to identify any taurine introgression and anoa, the wild buffalo (*Bubalus depressicornis*, OWB, n = 10) was included as an outgroup in the present study.

Genotype data from a total of 10 populations were included in subsequent analysis (Table 1). Mithun genotype data were merged with the other data into a single dataset by retaining markers shared between the HD and the 50k panels. The final data included total 209 samples and 38,587 SNPs. Markers and individuals were removed from the dataset using PLINK1.9 software^19^ if they did not have call rate of at least 95%. No filtering was done at this step for MAF, except SNPs monomorphic across all breeds. Summary statistics such as heterozygosity and inbreeding coefficient were computed and results are presented (Table 2). For principal component analysis, the dataset included total 144 samples keeping the maximum number of individuals set to 20 (Table 1). Population structure was inferred in two ways: principal component analyses (PCA) by *smartpca* from the Eigenstrat package^39^ and ADMIXTURE v. 1.23^18^. PCA uses an orthogonal transformation to convert a set of correlated variables into a set of linearly uncorrelated variables called principal components and maps individuals onto these major axes of variation. In contrast, ADMIXTURE provides maximum likelihood estimates of individual ancestries.

Phylogenetic analysis was carried out using Treemix^20^ and wild buffalo as an outgroup with between 0 and 5 migrations allowed. For Treemix analysis we used 21,939 SNPs polymorphic in mithun out of 38,587 SNPs used in PCA and ADMIXTURE analysis. 18 samples (3 Bali cattle, 9 mithuns from farm and 6 mithuns from field) showing admixture were removed from the Treemix analysis. Nodes robustness was estimated with 500 bootstrap replicates and plotted using the Treemix bootstrap function of BITE^40^. Admixture detection was done using the F3 statistics computed using threepop program from the Treemix software package.

### Ethics approval and consent of mithun owners

Approval of Institutional Animal Ethics Committee was obtained for this study including collection of mithun blood samples and DNA extraction from the Institute research farm. Blood samples of the mithun were collected from the farm and with the permission of the mithun owners from the field by qualified veterinarians.

### Data availability

Data supporting this paper were generated by ICAR-NRC on Mithun. The phenotype and genotype data are available with the Data Cell of the Institute and should be requested directly from the corresponding authors or the ICAR-NRC on Mithun.
